# Dual-Channel Reconstruction Network for Image Compressive Sensing

**DOI:** 10.3390/s19112549

**Published:** 2019-06-04

**Authors:** Zhongqiang Zhang, Dahua Gao, Xuemei Xie, Guangming Shi

**Affiliations:** School of Artificial Intelligence, Xidian University, Xi’an 710071, China; zqzhang6@stu.xidian.edu.cn (Z.Z.); xmxie@mail.xidian.edu.cn (X.X.); gmshi@xidian.edu.cn (G.S.)

**Keywords:** compressive sensing (CS), reconstruction, dual-channel

## Abstract

The existing compressive sensing (CS) reconstruction algorithms require enormous computation and reconstruction quality that is not satisfying. In this paper, we propose a novel Dual-Channel Reconstruction Network (DC-Net) module to build two CS reconstruction networks: the first one recovers an image from its traditional random under-sampling measurements (RDC-Net); the second one recovers an image from its CS measurements acquired by a fully connected measurement matrix (FDC-Net). Especially, the fully connected under-sampling method makes CS measurements represent original images more effectively. For the two proposed networks, we use a fully connected layer to recover a preliminary reconstructed image, which is a linear mapping from CS measurements to the preliminary reconstructed image. The DC-Net module is used to further improve the preliminary reconstructed image quality. In the DC-Net module, a residual block channel can improve reconstruction quality and dense block channel can expedite calculation, whose fusion can improve the reconstruction performance and reduce runtime simultaneously. Extensive experiments manifest that the two proposed networks outperform state-of-the-art CS reconstruction methods in PSNR and have excellent visual reconstruction effects.

## 1. Introduction

In the past decade, compressive sensing [1] theory has achieved great success in signal sampling paradigm because it can obtain high-quality recovery from CS measurements. Based on CS, several new imaging systems have been developed, such as single-pixel camera [2], compressive spectral imaging system [3], Hyperspectral imaging [4], high-speed video camera [5] and fast Magnetic Resonance Imaging (MRI) system [6].

For a given image $x\in R^{N}$, the CS linear measurements $y=\Phi x\in R^{M}$, where Φ is an M×N measurement matrix and M≪N. The original image has a sparse representation x=Ψs where Ψ is an N×N basis matrix. Compressive sensing is mainly concerned with the problem of recovering original image *x* from CS measurements *y*, which contains two kinds of methods: conventional iterative optimization strategies [7,8,9,10,11,12,13,14] and deep learning-based methods [15,16,17,18].

Early researchers have proposed some iterative algorithms such as matching pursuit [7], orthogonal matching pursuit (OMP) [8,9], iterative hard thresholding [11], iterative soft thresholding [12] and approximate message passing (AMP) [13,19]. However, these iterative algorithms are usually very slow to converge. To alleviate such difficulty, block-based CS methods have been proposed [20,21], although they still need expensive computation. Inspired by the great success of deep neural networks for computer vision tasks [22,23], learning-based CS reconstruction methods have been developed [15,16,17,18,24]. However, compared with the traditional CS methods, deep learning-based methods require additional training process, which brings the need for a training set. However, deep learning-based methods have faster reconstruction speed via a simple forward computing. Especially, the reconstruction performance and time complexity of existing learning-based methods are still not satisfying and can be further improved. In some learning-based methods such as SDA [15], DR${}^{2}$-Net [17], ConvCSNet [24] and ASRNet [25], especially, SDA and ConvCSNet directly obtain the CS measurements based on the whole image. This easily leads to an increase in computational complexity as the input image size increases. DR${}^{2}$-Net and ASRNet extract image patches from data set, while they need to build deeper models (enormous computation) to recover the original image. We have analysed this phenomenon: in the DR${}^{2}$-Net with single channel four residual blocks [17], authors compare the performance of fc1089-Res1, fc1089-Res2, fc1089-Res3, fc1089-Res4. We can easily find that fc1089-Res2 and fc1089-Res4 have similar performance at MRs = 0.01, 0.10, while the performance of fc1089-Res4 has not been greatly improved compared with fc1089-Res2. Especially, dense blocks [22] can strengthen feature propagation, encourage feature reuse, and substantially reduce the number of parameters. Therefore, we use one dense block to replace the two residual blocks of the fc1089-Res4. What is more, the connection mode is not a cascade but parallel, which can alleviate the vanishing-gradient problem to some extent. So we use dual-channel shallower network (less computation) of two residual blocks and one dense block to build the dual-channel reconstruction module. which can further improve the image reconstruction quality. Especially, a residual block channel can capture rich image features and improve the reconstruction performance. The dense block channel can expedite calculation because of its fewer parameters.

In this paper, we propose a novel dual-channel reconstruction module (DC-Net module) based on two residual blocks and one dense block, and we use this module to build two CS reconstruction networks: RDC-Net and FDC-Net. The first layer of the RDC-Net senses an input image by traditional Gaussian random matrix, while the first layer of the FDC-Net senses an input image by fully connected measurement matrix. The second layer of RDC-Net and FDC-Net is a fully connected layer to recover a preliminary reconstructed image. Then, DC-Net module is used to further improve the preliminary reconstructed image quality.

Extensive experiments show that the proposed networks can obtain better performance than the state-of-the-art CS reconstruction algorithms in terms of PSNR and visual effects. Our contributions can be summarized as follows:

- Unlike the deep-learning network with a very deep single-channel, we propose a novel shallow dual-channel reconstruction module for image compressive sensing reconstruction, in which each channel can extract different level features. It brings the better reconstruction quality.
- The proposed DC-Net module has two residual blocks and one dense block. Because the dense block has fewer parameters than residual block, the time complexity of the proposed method is lower than DR${}^{2}$-Net with four residual blocks.
- In our method, two residual blocks in one channel can obtain high level features and one dense block in another channel can obtain the low level features. Experiment results show both RDC-Net and FDC-Net have better robustness than DR${}^{2}$-Net.

## 2. Related Work

There are many traditional optimization algorithms [7,8,9,10,20,26,27] which are used to solve the CS reconstruction problem. AmitSatish Unde et al. proposed a reconstruction algorithm based on iterative re-weighted l1 norm minimization [20]. A.Metzler et al. proposed a denoising-based AMP framework (D-AMP), which integrated a wide class of denoiser within its iterations [14]. A. Metzler et al. also developed a novel neural network architecture that mimics the behavior of the denoising-based approximate message algorithm (LDAMP) [19]. Jin Tan et al. employed an adaptive Wiener filter as the image denoiser into AMP framework, called “AMP-Wiener”. They extended AMP-Wiener to three-dimension, called “AMP-3D-Wiener” for compressive hyperspectral imaging reconstruction problem [28]. Philip Schniter et al. integrated the D-AMP into auto-tuning method to form the D-VAMP [29]. E Tipping et al. presented an accelerated training algorithm for sparse bayesian models. They exploited a recent result concerning the properties of the marginal likelihood function to derive a ’constructive’ method for maximisation thereof [30]. Jiao Wu et al. proposed a stage-wise fast $l_{p}$-sparse Bayesian learning algorithm through integrating with a fast sequential learning scheme and a stage-wise strategy for CS reconstruction [31]. Thomas et al. proposed an iterative hard thresholding for compressed sensing [11]. Xiangming Meng et al. presented a unified Bayesian inference framework for generalized linear models (GLM), which iteratively reduced the GLM problem to a sequence of standard linear model (SLM) problems [32]. Jiang Zhu et al.proposed an approximate message passing-based generalized sparse Bayesian learning (AMP-Gr-SBL) algorithm to reduce the computation complexity of Gr-SBL algorithm [33]. Jun Fang et al. proposed a 2D pattern-coupled hierarchical Gaussian prior model to exploit the underlying block-sparse structure. This pattern-coupled hierarchical Gaussian prior model imposed a soft coupling mechanism among neighboring coefficients through their shared hyperparameters [34]. Mohammad Shekaramiz et al. proposed a new sparse Bayesian learning (SBL) method that incorporated a total variation-like prior as a measure of the overall clustering pattern in the solution [35]. Saman et al.presented an generative iterative thresholding algorithm for linear inverse problems with multi-constraints and its applications [26]. Bin Kang et al. proposed an efficient image fusion framework for multi-focus images based on compressed sensing. The new fusion framework consisted of three parts: image sampling, measurement fusion and image reconstruction. This novel fusion framework was capable of saving computational resource and enhancing the fusion result and was easy to implement [36]. Kezhi Li et al. proposed a new class of orthogonal circulant matrices built from deterministic sequences for convolution-based compressed sensing [37]. Nam Yul Yu et al. proposed to construct a filter with real-valued coefficients by taking the discrete Fourier transform of a decimated binary Sidelnikov sequence [38]. Weisheng Dong and Guangming Shi et al. presented a learning method for compressive image recovery. PAR models were first learned from training set and then used to regularize the compressive image recovery process [39]. However, the above algorithms suffer from serious time-consuming, which has become the bottleneck for the application of image compressive sensing.

In recent years, deep learning-based methods have shown promising performance in compressive image recovery [15,16,17,18,40]. Yu Simiao et al. proposed a conditional Generative Adversarial Networks-based deep learning framework for de-aliasing and reconstructing MRI images from highly undersampled data with great promise to accelerate the data acquisition process [41]. Guang Yang et al. provided a deep learning-based strategy for reconstruction of CS-MRI, and bridged a substantial gap between conventional non-learning methods working only on data from a single image, and prior knowledge from large training data sets [40]. Seitzer, Maximilian et al. proposed a hybrid method, in which a visual refinement component was learnt on top of an MSE loss-based reconstruction network [42]. Schlemper, Jo et al. proposed a novel cascaded convolution neural networks based on compressive sensing technique and explore its applicability to improve DT-CMR acquisitions [43]. The stacked denoising autoencoder (SDA) [15] considered the mapping from original signal to its measurement vector as one layer of the SDA. This kind of measurement method made SDA adapt its structure to the training set. However, it enhanced computational complexity along with the size of input image increased. Kulkarni et al. [16] proposed a block-based Network to realize the non-iterative image recovery. It took CS measurements of image block as input and output its corresponding reconstruction image block. DR${}^{2}$-Net [17] contained a linear mapping to recover a preliminary reconstructed image, in which residual blocks [23] could further improve the reconstruction quality. Xiaotong Lu and Weisheng Dong et al. [24] proposed a novel convolutional compressive sensing framework (ConvCSNet) based on deep convolutional neural network, which captured the image measurements by a convolutional operation.

Deep Residual Network: Lately, the deep residual network (ResNet) [23] had achieved promising performance on many computer vision tasks such as Image Recognition [23] and Image Denoising [44]. The ResNet introduces identity shortcut connections that directly pass the data flow to later layers compared with the traditional convolutional network. Therefore, we use the ResNet to avoid the loss attenuation caused by multiple non-linear transformations and ResNet consists of many residual blocks.

Densely Connected Network: Recently, the densely connected network (DensNet) [22] also obtained an enormous success in image detection, classification and semantic segmentation. Compared with the deep Residual Network [23], the DensNet introduces identity shortcuts to all layers, which makes a better use information of all features. Especially in reconstruction tasks, the architecture of DensNet can make comprehensive utilization of shallow detailed features to recover original image and DensNet consists of many dense blocks.

To further improve reconstruction quality and reduce runtime in CS reconstruction, in this paper, we use two residual blocks and one dense block to build a dual-channel reconstruction network module. This module can improve the image reconstruction quality and reduce time complexity simultaneously, which is used to build two CS reconstruction networks: the first one recovers original image from its CS measurements acquired by random Gaussian under-sampling measurements (RDC-Net) and the second one recovers original image from its CS measurements acquired by the fully connected measurement matrix (FDC-Net).

The remainder of this paper is organized as follows: In Section 3, we introduce dual-channel reconstruction network module and two kinds of reconstruction networks. In Section 4, we design extensive experiments to evaluate our proposed reconstruction networks. Finally, we conclude the paper in Section 5.

## 3. Network Architecture

**Figure 1:**
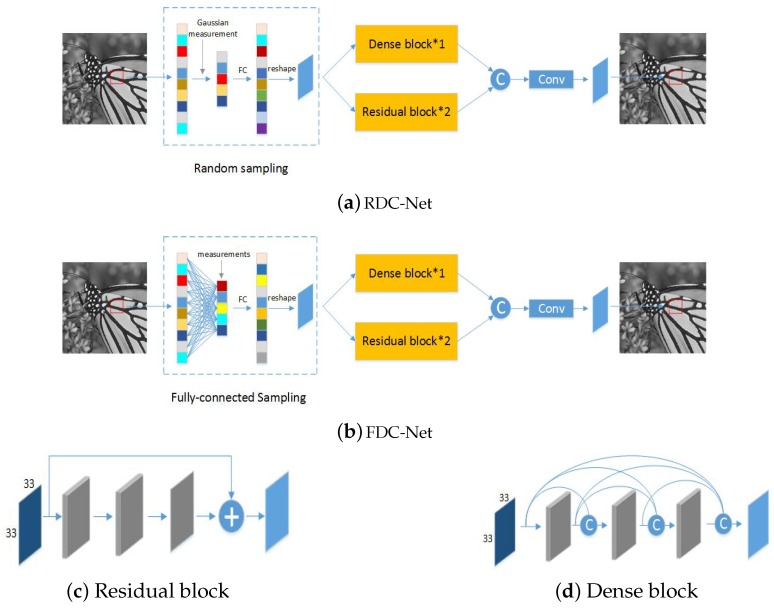
(**a**) The architecture of dual-channel reconstruction network with random measurement matrix (RDC-Net). (**b**) The architecture of dual-channel reconstruction network with fully connected measurement matrix (FDC-Net). (**c**) The structure of residual block. (**d**) The structure of dense block.

As shown in Figure 1. we propose two kinds of reconstruction networks: RDC-Net and FDC-Net. Firstly we introduce traditional random under-sampling, fully connected under-sampling approaches and preliminary reconstructed module. Then we discuss dual-channel reconstruction network module.

### 3.1. Under-Sampling and Preliminary Reconstruction

In the compressive sensing theory [1], there are some under-sampling approaches such as random Gaussian measurement [45], random Fourier measurement [46] and random Bernoulli measurement [47]. Random Gaussian measurement matrix is mostly used in CS theory and we also use Gaussian measurement matrix in RDC-Net.

(1) $$y=\left[\begin{array}{c} y_{1}\\ y_{2}\\ \vdots \\ y_{M} \end{array}\right]=\left[\begin{array}{cccccc} w_{11} & w_{12} & w_{13} & w_{14} & \cdots & w_{1N}\\ w_{21} & w_{22} & w_{23} & w_{24} & \cdots & w_{2N}\\ \vdots & \vdots & \vdots & \vdots & \ddots & \vdots \\ w_{M1} & w_{M2} & w_{M3} & w_{M4} & \cdots & w_{MN} \end{array}\right]\left[\begin{array}{c} x_{1}\\ x_{2}\\ x_{3}\\ x_{4}\\ \vdots \\ x_{N} \end{array}\right]$$

**Figure 2:**
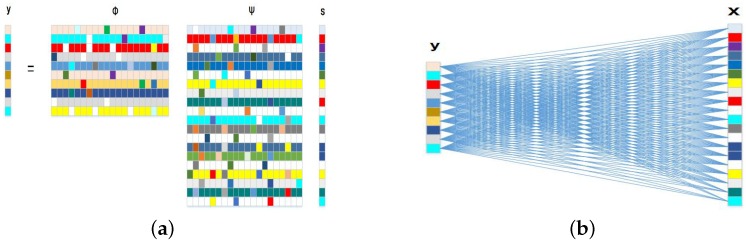
(**a**) Random Gaussian matrix is used as measurement matrix Φ. (**b**) The fully connected matrix is used as measurement matrix and the parameters are learned from training set.

In the FDC-Net, we use a fully connected layer (Figure 2b) as the measurement matrix to imitate the traditional under-sampling method in Figure 2a. In particular, such fully connected layer has no bias and activation function, and it learns a linear transformation from the original image to CS measurements. Both random Gaussian measurement matrix and learning measurement matrix have the similar mathematical formula as Equation (1). We expect that the learning measurement matrix (Equation (4)) is well adapted to the distribution of original image and denote this layer as $y^{f}=W_{1}\cdot x$, and the traditional Gaussian measurement method can also be expressed by $y^{r}=W_{2}\cdot x$, where $W_{1},W_{2}\in R^{M\times N}$ (M≪N) and $W_{2}$ conforms to the Gaussian distribution. Especially, *x* is the original image and $y^{r},y^{f}$ are the corresponding CS measurements of RDC-Net and FDC-Net, respectively.

Afterwards, we use a fully connected layer to recover a preliminary reconstructed image $x_{c}^{*}$. We denote the preliminary reconstructed module and the corresponding parameters as $f\left(\cdot \right)$ and $\Omega_{c}^{p}$ respectively, where $c\in \left\{\mathrm{RDC}\text{-}\mathrm{Net},\mathrm{FDC}\text{-}\mathrm{Net}\right\}$, *p* represents the preliminary reconstructed module. The preliminary reconstructed image can be expressed by:

(2) $$\begin{array}{c} x_{c}^{*}=f\left(y^{c},\;\Omega_{c}^{p}\right) \end{array}$$

and mean squared error (MSE) is used as the loss function for the training set:

(3) $$\begin{array}{c} L\left(\{\Omega_{c}^{p}\}\right)=\frac{1}{M}\sum_{i=1}^{M}{∥f\left(y_{i}^{c},\;\Omega_{c}^{p}\right)-x_{i}∥}_{2}^{2} \end{array}$$

(4) $$\begin{array}{c} W_{1}=\underset{w_{1}}{\arg\min}\;\frac{1}{M}\sum_{i=1}^{M}{∥f\left(W_{1}\cdot x_{i},\;\Omega_{f}^{p}\right)-x_{i}∥}_{2}^{2} \end{array}$$

where $M,W_{1}$ represent the number of training samples and learning measurement matrix respectively. Back propagation [48] algorithm is used to minimize the loss function defined in Equation (3).

### 3.2. Dual-Channel Network Module

In Section 3.1, we only obtain a preliminary reconstructed image for the reason that it is not easy to get an exact solution in preliminary reconstructed module. Then, the dual-channel network module is used to further improve the reconstruction quality. In this paper, two residual blocks and one dense block are used as one channel separately, and they are fused to build a dual-channel network module. We firstly make a brief introduction to residual block and dense block.

Compared with the traditional convolutional network, the main difference of the residual network is that it introduces identity connections that directly pass the data flow to later layers. Given an input χ, we expect the output of a few stacked layers in network as $T\left(\chi \right)$. However, it takes great expense to optimize $T\left(\cdot \right)$ in traditional convolutional network. In [23], K. He et al. proposed to approximate the residual value between $T\left(\chi \right)$ and χ with the stacked layers. The residual block (Figure 1c) can be expressed by

(5) $$\begin{array}{c} F\left(\chi \right)=T\left(\chi \right)-\chi \end{array}$$

In [22], Gao Huang et al. proposed the Dense Convolutional Network (DensNet) for many computer vision tasks. The traditional convolutional networks with L layers have L connections. While the DensNet has $\frac{L(L+1)}{2}$ direct connections, which strengthens feature propagation, encourages feature reuse and enormously reduces the number of parameters. This kind of network is very useful in the compressive sensing field. In dense block (Figure 1d), for each layer, all preceding feature maps are used as its inputs, and its own feature maps are also used as inputs into all subsequent layers. In other words, it means the *m*th layer can connect the feature maps of all preceding layers $\chi_{0},\chi_{1},\ldots ,\chi_{m-1}$ as inputs:

(6) $$\begin{array}{c} \chi_{m}=\Gamma_{m}\left(\left[\chi_{0},\chi_{1},\ldots ,\chi_{m-1}\right]\right) \end{array}$$

where $\left[\chi_{0},\chi_{1},\ldots ,\chi_{m-1}\right]$ denotes the concatenate operation of the feature maps in layers 0,1,…,m−1. $\Gamma_{m}\left(.\right)$ can be regarded as a composite function of four consecutive operations: batch normalization (BN), scale layer, rectified linear unit (ReLU) and convolution (Conv).

We denote the dual-channel network module as H(χ) that contains two residual blocks and one dense block, which can be expressed by

(7) $$\begin{array}{c} H(\chi )=2⊗F(\chi )⊕1⊗\Gamma (\chi ) \end{array}$$

where the symbol ⊗ represents cascaded operation and ⊕ represents parallel operation between one dense block and two residual blocks.

In this paper, H(χ) takes $x_{c}^{*}$ as input and outputs final reconstruction result, which can be represented as:

(8) $$\begin{array}{c} \hat{x_{i}^{c}}=H_{c}\left(x_{c}^{*},\;\;\Omega_{c}^{d}\right) \end{array}$$

where *d* represents the dual-channel network module and the $\Omega_{c}^{d}$ represents the parameters of dual-channel network module. The loss function of the proposed networks can be expressed by

(9) $$\begin{array}{c} \begin{array}{cc} L\left(\{\Omega_{c}^{p},\;\Omega_{c}^{d}\right\}) & =\frac{1}{M}\sum_{i=1}^{M}{∥\overset{\;}{\hat{x_{i}^{c}}}\;-x_{i}∥}_{2}^{2}\\ & =\frac{1}{M}\sum_{i=1}^{M}{∥H_{c}\left(x_{c}^{*},\;\Omega_{c}^{d}\right)-x_{i}∥}_{2}^{2}\\ & =\frac{1}{M}\sum_{i=1}^{M}{∥H_{c}\left(f\left(y_{i}^{c},\;\Omega_{c}^{p}\right),\;\Omega_{c}^{d}\right)-x_{i}∥}_{2}^{2} \end{array} \end{array}$$

### 3.3. Architecture

The architectures of proposed networks are shown in Figure 1. In the RDC-Net (Figure 1a), we take the 33 × 33 sized image block as input and acquire CS measurements by traditional random measurement matrix. In the FDC-Net (Figure 1b), we take the same sized image block as input and acquire CS measurements by fully connected measurement matrix. With the CS measurements, the preliminary reconstructed image can be realized via a fully connected layer. Then, the dual-channel reconstruction network module H(χ) takes the preliminary reconstructed image as input and outputs the corresponding higher quality image. Finally, the BM3D [49] is used to remove the artifacts caused by block-wise processing.

## 4. Experiments

In this section, we perform a multitude of experiments to test the performance of the proposed networks on the Caffe [50] platform. Our computer is equipped with intel core i7-6700 with frequency of 3.4 GHz and Nvidia GeForce GTX 1080Ti, and the network framework runs on the ubuntu system.

### 4.1. Training Data

For a fair comparison, the same dataset [16] is used to generate the training data and test data. We use the luminance component of the images and extract 33 × 33 sized image patches with stride 14 from 91 images [16] as training set. We also use the luminance component of the images and extract 33 × 33 sized image patches with stride 14 from 5 images [16] as test images. Both RDC-Net and FDC-Net use the same dataset and are trained with different MRs = 0.01, 0.04, 0.10 and 0.25. Especially, we take about 8 h to train the proposed networks.

### 4.2. Training Strategy

The training procedure of RDC-Net and FDC-Net consists of two steps. In the first step, we train preliminary reconstructed module with a slightly big learning rate to obtain the preliminary reconstructed image and parameters of $\Omega_{c}^{p}$. The maximum number of iterations, the learning rate, the step size, the batch size and the gamma are set as 800,000, 0.001, 200,000, 128 and 0.5, respectively. The second step is to optimize preliminary reconstruction module and DC-Net module with a gradually decline learning rate and updates parameters of $\Omega_{c}^{p}$ and $\Omega_{c}^{d}$. Especially, the maximum number of iterations, the learning rate, the decay rate, the decay steps and the batch size are set as 200,000, 0.0001, 0.98, 1000 and 64.

### 4.3. Comparison with Other Methods

In this part, we compare two proposed networks with existing methods such as NLR-CS [51], D-AMP [14], TVAL3 [10], ReconNet [16], SDA [15], DR${}^{2}$-Net [17] and ConvCSNet [24]. In particular, NLR-CS, TVAL3, D-AMP, ReconNet, DR${}^{2}$-Net, CSRNet and RDC-Net obtain the CS measurements by traditional random measurement matrix. SDA [15], ConvCSNet [24], ASRNet and FDC-Net obtain CS measurements by learning-based approaches. The results of TVAL3, NLR-CS, D-AMP, ReconNet and DR${}^{2}$-Net are from the code presented by the respective authors on their websites. Especially, the results of SDA are from our own reproduction. The results of CSRNet and ASRNet refer to the paper [25]. In the training stage, we use the default parameters to train these networks many times to get the many test models. Then we use these test models to obtain reconstruction results. In this paper, we choose PSNR and SSIM as the evaluation criterions. The related experiment results are summarized in Table 1 and Table 2, where the best results are highlighted in bold.

**Figure 3:**
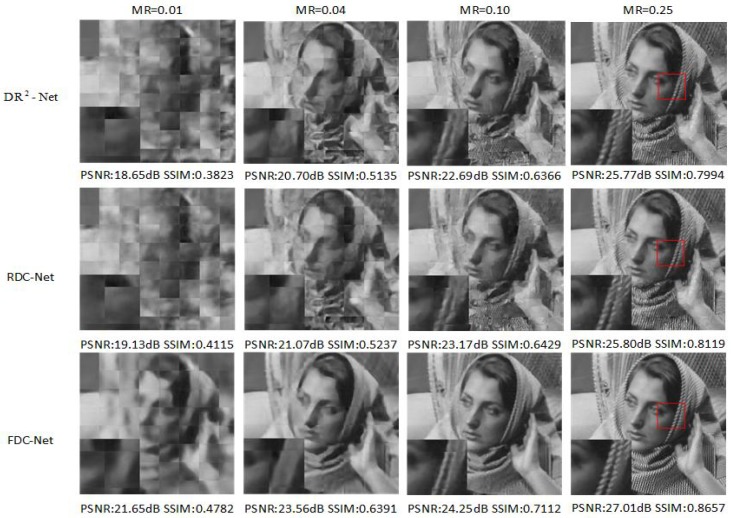
Barbara image reconstruction results from different networks. We can obviously find that two proposed networks obtain the excellent reconstruction performance and FDC-Net has better visual effects than RDC-Net, DR${}^{2}$-Net.

**Figure 4:**
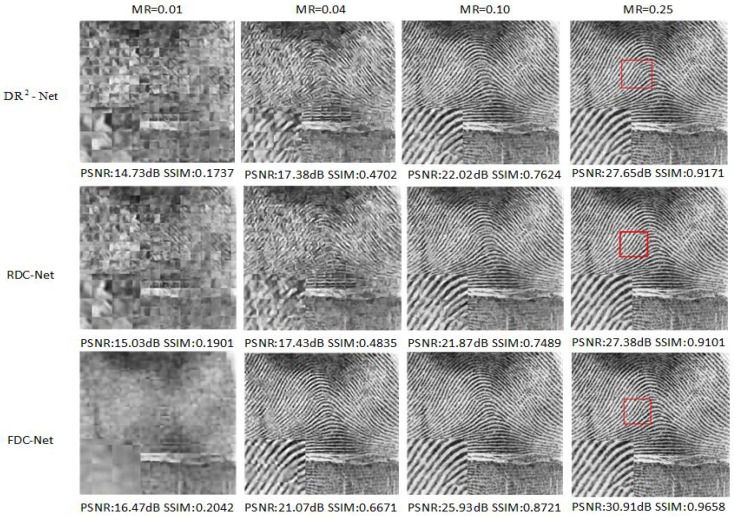
Fingerprint image reconstruction results from different networks. We can obviously find that two proposed networks obtain the excellent reconstruction performance and FDC-Net has better visual effects than RDC-Net, DR${}^{2}$-Net.

**Figure 5:**
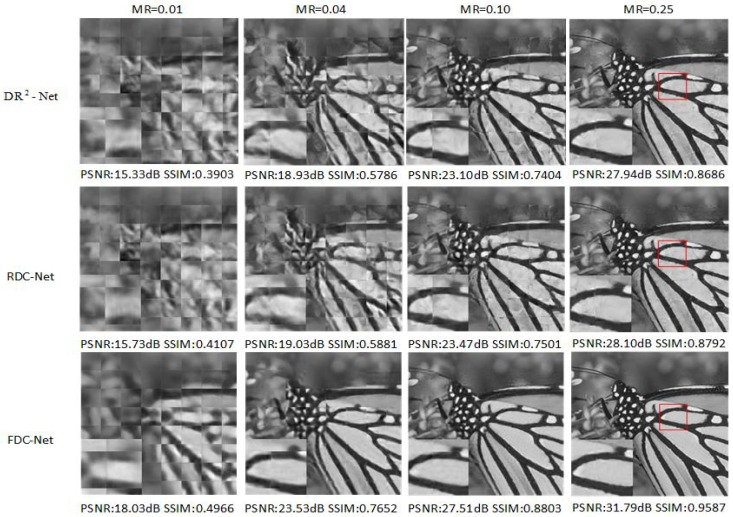
Monarch image reconstruction results from different networks. We can obviously find that two proposed networks obtain the excellent reconstruction performance and FDC-Net has better visual effects than RDC-Net, DR${}^{2}$-Net.

As shown in Table 1, RDC-Net obtains the higher mean PSNR values than other methods at MRs = 0.10, 0.25. However, in some test images (e.g., barbara, Fingerprint, Flinstones), other reconstruction methods (NLR-CS or DR${}^{2}$-Net) obtain slightly higher reconstruction quality, and we also compare the reconstruction performance of FDC-Net, SDA, ConvCS-Net and ASRNet in Table 2. It is obvious that FDC-Net outperforms other methods at measurement rates 0.01, 0.04, 0.10 and 0.25. Especially at MR = 0.25. FDC-Net obtains 2.3 dB improvement than the second highest value. In Figure 3, Figure 4 and Figure 5, we compare the visual reconstruction results among FDC-Net, RDC-Net and DR${}^{2}$-Net. We can easily find that our reconstruction results have better visual effects. For example, Figure 4 is a fingerprint image. Our visual reconstruction results have a clearer texture, clean areas and sharp edges than DR${}^{2}$-Net in the enlarged patches at four MRs, while the visual reconstruction results of DR${}^{2}$-Net have blurred textures and confusing areas.

### 4.4. Evaluation on Different Network Architectures

In order to evaluate the effectiveness of our main model, FDC-Net, we design other different network architectures such as single channel networks (One-densblock and Two-resblocks)and dual-channel networks (one-resblock + one-densblock, two-resblocks + two-densblock, three-resblocks + one-densblock). “One-densblock” means that we use the dense block channel (Figure 1b) to recover the image from CS measurements. “Two-resblocks” means that we use the residual block channel (Figure 1b) to recover the image. “one-resblock + one-densblock”, “two-resblocks + two-densblock” and “three-resblocks + one-densblock” represent that we use one residual block and one dense block, two residual blocks and two dense blocks, three residual blocks and one dense block to improve the preliminary reconstructed image quality respectively. The relevant results are summarized in Table 3, where the best results are highlighted in bold.

As shown in Table 3, it is obvious that FDC-Net outperforms other networks at MRs = 0.04, 0.10, 0.25. When we only use one channel module (one-densblock or two-resblocks) to recover the original image from its CS measurements, the reconstruction results are good. But we combine two channel modules, FDC-Net obtains obviously outstanding performances, which is probably because the residual block channel can improve reconstruction quality and dense block channel can expedite calculation. One-resblock + one-densblock, three-resblocks + one-densblock and two-resblocks + two-densblocks all obtain outstanding performance. Although the three-resblocks + one-densblocks obtains higher PSNR than FDC-Net at MR = 0.01, it increases the time complexity and has lower PSNR than FDC-Net at MRs = 0.04, 0.10, 0.25. Therefore, we use the two residual blocks and one dense block to build the dual-channel reconstruction module.

### 4.5. Robustness to Noise

**Figure 6:**
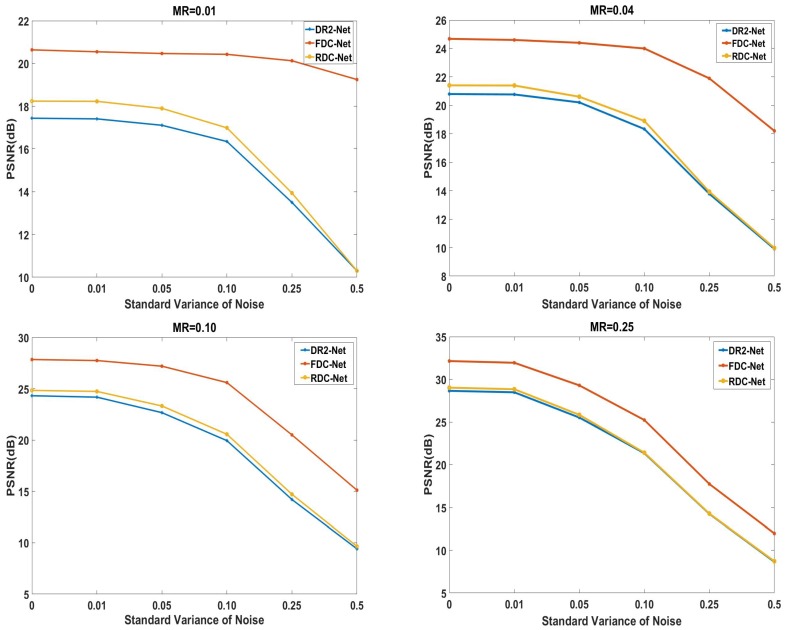
Robustness comparison to different Gaussian noise among the different networks.

To show the robustness of proposed networks to noise, we perform reconstruction experiments under the presence of measurement noise. The standard Gaussian noise is added to CS measurements of test set. We add five levels of noise corresponding to δ= 0.01, 0.05, 0.10, 0.25 and 0.5, where δ is the standard variance for the Gaussian noise. Then, two proposed networks trained on the noiseless CS measurements take the noisy CS measurements as input and output the reconstruction images. Here, we mainly compare the three algorithms: DR${}^{2}$-Net, RDC-Net, FDC-Net. The related results are summarized in Figure 6.

From Figure 6, it is obvious that two proposed networks mostly outperform the DR${}^{2}$-Net for δ= 0.01, 0.05, 0.10, 0.25 and 0.5 at four MRs. Especially, the decay of FDC-Net’s performance is slower than DR${}^{2}$-Net’s at MRs = 0.01, 0.04, which indicates that our FDC-Net have outstanding robustness at low measurement rates.

### 4.6. Evaluation on ImageNet Val Dataset

To testify the scalability of proposed networks, we also perform reconstruction experiments between two proposed networks with DR${}^{2}$-Net on the large-scale ImageNet val dataset [52] and it includes 50,000 images of 1000 classes. The experimental results are shown in Table 4, where the best results are highlighted in bold.

As shown in Table 4, the two proposed networks obtain better performances than DR${}^{2}$-Net at four MRs. Especially at MR = 0.25, RDC-Net, FDC-Net achieves nearly 3 dB and 5 dB improvement over DR${}^{2}$-Net, respectively, which indicates that our proposed networks have better generalization ability than DR${}^{2}$-Net.

### 4.7. Time Complexity and Network Convergence

In this paper, we also perform the time complexity experiments between two proposed algorithms and DR${}^{2}$-Net. The related results are shown in Table 5, where the best results are highlighted in bold.

From Table 5, we can observe that two proposed networks have slightly less runtime than DR${}^{2}$-Net, and FDC-Net gains best results, which is helpful for CS real-time applications.

**Figure 7:**
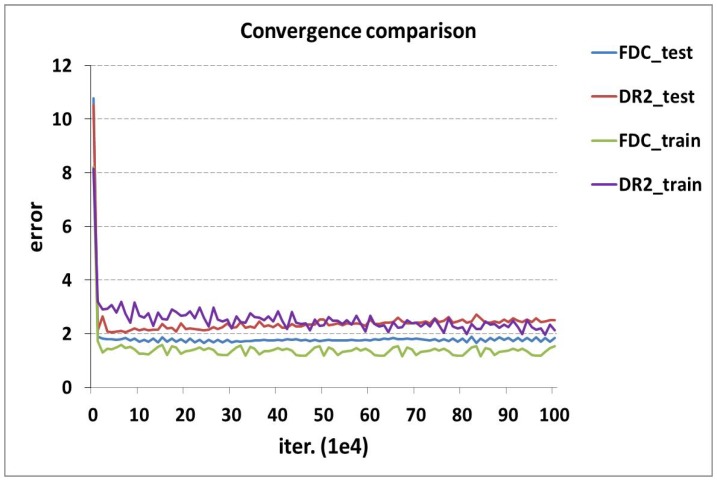
Convergence comparison between FDC-Net and DR${}^{2}$-Net. It is obvious that our FDC-Net has smaller training error and test error than DR${}^{2}$-Net.

In order to further demostrate that our proposed networks have better convergence performance than DR${}^{2}$-Net, we perform a convergence experiment between FDC-Net and DR${}^{2}$-Net at MR = 0.04. Figure 7 shows that training error and test error of FDC-Net are smaller than DR${}^{2}$-Net, which demonstrates that our network is easier to converge than DR${}^{2}$-Net.

## 5. Conclusions

Inspired by the fact that deep learning-based methods can improve reconstruction performance and enormously reduce computation compared to traditional iterative reconstruction algorithms, we propose a novel dual-channel reconstruction network module (DC-Net module) to build two CS reconstruction networks: the first one recovers an image from its traditional random under-sampling measurements (RDC-Net); the second one recovers an image from its CS measurements acquired by a fully connected measurement matrix (FDC-Net). Especially, DC-Net module consists of one dense block and two residual blocks. We use a fully connected layer to obtain a preliminary reconstructed image, and DC-Net module is used to further improve the preliminary reconstructed image quality. Extensive experiments show that our networks outperform the state-of-the-art CS algorithms in both PSNR and visual quality. Moreover, our networks also have outstanding robustness and lower time complexity.

## Figures and Tables

**Table 1:** Reconstruction results for test images through different algorithms at different measurement rates. “Mean” is the mean value among all test images.

Image Name	Methods	PSNR (without Using BM3D/with Using BM3D)							
		MR = 0.01		MR = 0.04		MR = 0.10		MR = 0.25	
Barbara	TVAL3	11.94	11.96	18.97	18.99	21.85	22.23	24.21	24.26
	NLR-CS	5.50	5.86	11.08	11.56	14.80	14.84	28.01	28.00
	D-AMP	5.48	5.51	16.37	16.37	21.23	21.24	25.08	25.96
	ReconNet	18.61	19.07	20.38	21.20	21.90	22.51	23.20	23.55
	DR${}^{2}$-Net	18.65	19.10	20.69	21.31	22.69	22.84	25.77	25.99
	CSRNet	19.10	19.21	21.27	21.49	22.94	22.95	26.17	26.34
	RDC-Net	19.13	19.22	21.07	21.18	23.17	23.21	25.80	25.91
Fingerprint	TVAL3	10.35	10.37	16.03	16.07	18.68	18.71	22.71	22.68
	NLR-CS	4.85	5.19	9.67	10.10	12.80	12.84	23.51	23.52
	D-AMP	4.66	4.74	13.83	14.00	17.13	17.14	25.18	24.15
	ReconNet	14.82	14.88	16.91	16.96	20.75	20.96	25.57	25.14
	DR${}^{2}$-Net	14.73	14.95	17.38	17.47	22.02	22.44	27.65	27.76
	CSRNet	15.11	15.18	17.59	17.68	21.64	21.91	27.22	27.49
	RDC-Net	15.03	15.05	17.43	17.45	21.87	21.89	27.38	27.91
Flinstones	TVAL3	9.75	9.78	14.87	14.91	18.89	18.93	24.06	24.08
	NLR-CS	4.45	4.76	8.98	9.26	12.15	12.24	22.41	22.66
	D-AMP	4.33	4.35	12.94	13.07	16.94	16.86	25.02	24.46
	ReconNet	13.96	14.07	16.31	16.56	18.92	19.20	22.46	22.60
	DR${}^{2}$-Net	14.00	14.18	16.94	17.06	21.08	21.45	26.19	26.79
	CSRNet	14.32	14.39	17.29	17.41	20.52	20.82	25.46	25.47
	RDC-Net	14.29	14.51	17.15	17.38	21.00	21.88	25.94	26.08
Lena	TVAL3	11.87	11.91	19.47	19.53	24.17	24.21	28.68	28.72
	NLR-CS	5.96	6.26	11.62	11.98	15.31	15.34	29.39	29.67
	D-AMP	5.73	5.96	16.53	16.87	22.53	22.54	28.00	27.46
	ReconNet	17.87	18.07	21.28	21.83	23.83	24.51	26.52	26.55
	DR${}^{2}$-Net	17.97	18.43	22.13	22.73	25.38	25.77	29.42	29.64
	CSRNet	19.11	19.20	22.89	23.17	25.72	25.97	29.55	29.70
	RDC-Net	18.69	18.96	23.17	23.37	26.19	26.57	29.78	29.97
Monarch	TVAL3	11.09	11.12	16.74	16.75	21.16	21.16	27.75	27.77
	NLR-CS	6.38	6.76	11.62	11.98	14.60	14.67	25.91	26.10
	D-AMP	6.21	6.21	14.57	14.57	19.00	19.00	26.39	26.56
	ReconNet	15.39	15.47	18.18	18.33	21.10	22.51	24.32	25.05
	DR${}^{2}$-Net	15.33	15.50	18.93	19.23	23.10	23.54	27.94	28.30
	CSRNet	15.42	15.46	19.41	19.60	22.99	23.25	27.98	28.37
	RDC-Net	15.73	15.97	19.03	19.31	23.47	23.61	28.10	29.52
Parrot	TVAL3	11.44	11.46	18.88	18.91	23.13	23.15	27.18	27.24
	NLR-CS	5.12	5.44	10.60	10.92	14.14	14.18	26.53	26.72
	D-AMP	5.08	5.08	15.78	15.78	21.63	21.63	26.88	26.99
	ReconNet	17.61	18.31	20.27	21.06	22.63	23.25	25.59	26.22
	DR${}^{2}$-Net	18.01	18.41	21.16	21.86	23.95	24.32	28.72	29.10
	CSRNet	19.50	19.61	22.16	22.31	24.79	25.01	28.86	29.05
	RDC-Net	19.27	19.71	21.86	21.98	24.45	24.98	28.94	29.01
Boats	TVAL3	11.86	11.87	19.21	19.21	23.85	23.86	28.81	28.81
	NLR-CS	5.38	5.73	10.77	11.22	14.83	14.86	29.11	29.25
	D-AMP	5.34	5.35	16.01	16.01	21.95	21.95	29.26	29.26
	ReconNet	18.49	18.87	21.38	21.62	24.15	24.21	27.30	27.35
	DR${}^{2}$-Net	18.67	18.96	22.11	22.50	25.58	25.91	30.09	30.30
	CSRNet	18.99	19.09	22.38	**22.55**	25.65	25.80	30.14	30.36
	RDC-Net	19.16	19.32	22.20	22.35	25.91	26.08	30.18	30.35
Cameraman	TVAL3	11.97	11.98	18.30	18.33	21.91	21.92	25.69	25.70
	NLR-CS	5.98	6.36	11.04	11.46	14.18	14.22	24.88	24.97
	D-AMP	5.64	5.65	15.12	15.12	20.35	20.35	24.42	24.56
	ReconNet	17.11	17.49	19.28	19.73	21.29	21.67	23.16	23.61
	DR${}^{2}$-Net	17.08	17.34	19.84	20.31	22.46	22.76	25.61	25.91
	CSRNet	17.75	17.90	20.23	20.38	22.29	22.53	25.85	26.15
	RDC-Net	17.95	18.21	20.38	20.61	22.93	23.11	25.96	26.17
Foreman	TVAL3	10.98	11.02	20.64	20.65	28.69	28.74	35.41	35.55
	NLR-CS	3.92	4.26	9.08	9.46	13.53	13.54	35.73	35.91
	D-AMP	3.84	3.84	16.27	16.31	25.50	25.53	35.45	35.06
	ReconNet	20.04	20.33	23.72	24.61	27.10	28.58	29.47	30.79
	DR${}^{2}$-Net	20.59	21.08	25.34	26.32	29.20	30.18	33.53	34.28
	CSRNet	23.12	**23.32**	27.78	28.18	30.96	31.35	34.89	35.10
	RDC-Net	22.98	23.07	27.27	27.29	31.29	31.61	35.11	35.31
House	TVAL3	11.86	11.90	20.94	20.96	26.29	26.33	32.09	32.14
	NLR-CS	4.96	5.26	10.66	11.08	14.77	14.80	34.20	34.21
	D-AMP	5.00	5.01	16.91	16.37	24.83	24.73	33.64	32.96
	ReconNet	19.31	19.52	22.57	23.20	26.69	26.70	28.47	29.20
	DR${}^{2}$-Net	19.61	19.99	23.91	24.70	27.52	28.42	31.82	32.52
	CSRNet	20.67	20.79	24.55	24.85	28.24	28.68	32.46	33.05
	RDC-Net	20.68	20.87	24.89	24.92	28.57	28.81	32.87	33.07
Peppers	TVAL3	11.35	11.37	18.21	18.23	22.64	22.65	29.61	29.65
	NLR-CS	5.76	6.11	11.38	11.81	14.94	14.99	28.89	29.24
	D-AMP	5.79	5.84	16.17	16.46	21.33	21.38	**29.88**	28.96
	ReconNet	16.83	16.98	19.57	20.00	22.15	22.68	24.77	25.15
	DR${}^{2}$-Net	16.90	17.11	20.32	20.75	23.72	24.26	28.48	29.11
	CSRNet	17.61	17.67	21.18	21.51	24.35	24.65	28.58	29.19
	RDC-Net	17.69	17.71	21.03	21.21	24.39	24.64	29.27	29.97
Mean	TVAL3	11.31	11.34	18.39	18.41	22.84	22.90	27.84	27.87
	NLR-CS	5.30	5.64	10.59	10.98	14.19	14.23	28.05	28.20
	D-AMP	5.19	5.23	15.50	15.54	21.13	21.12	28.11	27.85
	ReconNet	17.28	17.55	19.99	20.46	22.77	23.34	25.53	25.93
	DR${}^{2}$-Net	17.41	17.73	20.80	21.29	24.25	24.72	28.66	29.06
	CSRNet	18.25	18.35	21.52	21.74	24.55	24.81	28.83	29.11
	RDC-Net	18.24	18.42	21.41	21.55	24.84	25.13	29.03	29.39

**Table 2:** Reconstruction results for test images through different algorithms at different measurement rates. “Mean” is the mean value among all test images.

Image Name	Methods	PSNR (without Using BM3D/with Using BM3D)							
		MR = 0.01		MR = 0.04		MR = 0.10		MR = 0.25	
Barbara	SDA	18.59	18.76	20.49	20.86	22.17	22.39	23.19	23.21
	ConvCSNet	18.14	18.35	20.85	21.00	22.95	23.01	25.85	25.98
	ASRNet	21.40	21.52	23.48	23.54	**24.34**	24.35	26.30	26.43
	FDC-Net	21.65	21.71	23.56	23.71	24.25	24.52	27.91	28.00
Fingerprint	SDA	14.81	14.82	16.85	16.87	20.29	20.32	24.29	24.21
	ConvCSNet	14.54	14.82	18.44	18.71	19.76	20.11	28.00	28.11
	ASRNet	16.20	16.21	20.98	21.45	26.25	26.83	28.82	29.23
	FDC-Net	16.47	16.52	21.07	21.11	25.93	26.11	30.91	31.08
Flinstones	SDA	13.91	13.96	16.21	16.10	18.40	18.21	20.88	20.21
	ConvCSNet	15.04	15.32	17.22	17.58	19.49	19.82	26.42	26.53
	ASRNet	16.30	16.39	19.78	20.08	24.01	24.56	26.93	27.40
	FDC-Net	16.43	16.49	20.29	20.54	24.42	24.55	28.81	28.91
Lena	SDA	17.84	17.95	21.17	21.56	23.81	24.16	25.87	25.70
	ConvCSNet	17.97	18.16	21.78	22.08	25.27	25.61	27.11	27.32
	ASRNet	21.74	21.93	25.74	25.93	28.54	28.78	30.65	30.89
	FDC-Net	21.67	21.71	26.25	26.38	28.85	28.93	32.69	32.91
Monarch	SDA	15.31	15.38	18.11	18.19	20.95	21.04	23.54	23.32
	ConvCSNet	16.31	16.81	18.92	19.18	21.76	22.01	26.59	26.71
	ASRNet	17.74	17.85	23.23	23.49	27.17	27.50	29.29	29.60
	FDC-Net	18.03	18.46	23.53	23.52	27.51	27.83	31.79	31.97
Parrot	SDA	17.71	17.89	20.37	20.67	22.14	22.35	24.48	24.37
	ConvCSNet	17.86	18.15	20.55	21.18	24.41	24.85	26.26	26.38
	ASRNet	21.87	22.01	24.52	24.67	27.68	27.85	29.61	29.80
	FDC-Net	22.09	22.25	24.50	24.74	27.68	27.84	31.76	31.94
Boats	SDA	18.55	18.68	21.29	21.54	24.01	24.18	26.56	26.24
	ConvCSNet	18.11	18.39	21.81	22.08	24.82	25.31	27.86	27.98
	ASRNet	21.53	21.69	25.52	25.72	28.86	29.17	31.28	31.64
	FDC-Net	21.39	21.50	25.77	26.00	29.08	29.18	33.95	33.97
Cameraman	SDA	17.06	17.19	19.31	19.56	21.15	21.30	22.77	22.64
	ConvCSNet	17.61	17.92	19.40	20.01	22.31	22.69	25.15	25.26
	ASRNet	19.77	19.89	22.74	22.88	25.00	25.13	26.46	26.66
	FDC-Net	20.13	20.21	22.94	23.08	25.28	25.31	28.97	29.04
Foreman	SDA	20.08	20.24	23.62	24.09	26.43	27.16	28.40	28.91
	ConvCSNet	19.09	19.54	22.46	22.81	25.97	26.11	30.39	30.81
	ASRNet	25.77	26.14	30.56	30.78	33.79	34.09	35.85	36.19
	FDC-Net	25.87	25.92	31.08	31.18	34.51	34.71	38.25	38.39
House	SDA	19.45	19.59	22.51	22.94	25.41	26.07	27.65	27.86
	ConvCSNet	18.40	18.82	22.22	22.71	26.46	26.51	26.76	26.98
	ASRNet	23.13	23.31	27.82	28.21	31.47	31.87	33.44	33.84
	FDC-Net	23.08	23.14	28.03	28.21	31.67	31.79	35.75	35.98
Peppers	SDA	16.93	17.04	19.63	19.89	22.10	22.35	24.31	24.15
	ConvCSNet	17.69	18.01	20.76	21.08	23.12	23.66	26.26	26.51
	ASRNet	20.17	20.33	24.03	24.32	27.03	27.37	29.72	30.18
	FDC-Net	20.21	20.56	24.41	24.72	27.10	27.21	32.81	32.92
Mean	SDA	17.29	17.41	19.96	20.21	22.43	22.68	24.72	24.55
	ConvCSNet	17.34	17.66	20.40	20.77	23.30	23.61	26.97	27.14
	ASRNet	20.51	20.66	24.40	24.65	27.65	27.96	29.85	30.17
	FDC-Net	20.64	20.77	24.68	24.83	27.84	28.00	32.15	32.28

**Table 3:** Performance comparison among different network architectures.

Models	PSNR (without Using BM3D/with Using BM3D)							
	MR = 0.01		MR = 0.04		MR = 0.10		MR = 0.25	
One-densblock	20.41	20.52	24.17	24.82	27.29	27.55	30.29	30.85
Two-resblocks	20.37	20.50	24.41	24.72	27.52	27.83	30.57	30.99
one-resblock+one-densblock	20.43	20.48	24.43	24.75	27.50	27.78	30.47	30.98
two-resblocks+two-densblock	20.60	20.78	24.57	24.80	27.54	27.76	31.64	32.06
three-resblocks+one-densblock	20.66	20.83	24.60	24.89	27.49	27.68	31.55	31.81
FDC-Net	20.64	20.82	24.68	24.97	27.84	28.27	32.15	32.42

**Table 4:** The PSNR value of different networks on ImageNet Val dataset.

Models	MR = 0.01	MR = 0.04	MR = 0.10	MR = 0.25
DR${}^{2}$-Net	23.27	25.90	27.78	29.10
RDC-Net	23.87	26.92	29.76	32.07
FDC-Net	25.76	29.11	31.32	34.05

**Table 5:** Time (s) for reconstructing a single 512 × 512 image.

Models	MR = 0.01	MR = 0.04	MR = 0.10	MR = 0.25
DR${}^{2}$-Net	0.0686	0.0676	0.0680	0.0678
RDC-Net	0.0590	0.0591	0.0595	0.0591
FDC-Net	0.0570	0.0566	0.0579	0.0571
